# Complementary Amplicon-Based Genomic Approaches for the Study of Fungal Communities in Humans

**DOI:** 10.1371/journal.pone.0116705

**Published:** 2015-02-23

**Authors:** Timothy Heisel, Heather Podgorski, Christopher M. Staley, Dan Knights, Michael J. Sadowsky, Cheryl A. Gale

**Affiliations:** 1 Department of Pediatrics, University of Minnesota, Minneapolis, MN, 55454, United States of America; 2 Biotechnology Institute, University of Minnesota, St. Paul, MN, 55108, United States of America; 3 Department of Computer Science and Engineering, University of Minnesota, Minneapolis, MN, 55455, United States of America; 4 Department of Soil, Water, and Climate, University of Minnesota, St. Paul, MN, 55108, United States of America; 5 Department of Genetics, Cell Biology and Development, University of Minnesota, Minneapolis, MN, 55455, United States of America; University of Aberdeen, UNITED KINGDOM

## Abstract

Recent studies highlight the importance of intestinal fungal microbiota in the development of human disease. Infants, in particular, are an important population in which to study intestinal microbiomes because microbial community structure and dynamics during this formative window of life have the potential to influence host immunity and metabolism. When compared to bacteria, much less is known about the early development of human fungal communities, owing partly to their lower abundance and the relative lack of established molecular and taxonomic tools for their study. Herein, we describe the development, validation, and use of complementary amplicon-based genomic strategies to characterize infant fungal communities and provide quantitative information about *Candida*, an important fungal genus with respect to intestinal colonization and human disease. Fungal communities were characterized from 11 infant fecal samples using primers that target the internal transcribed spacer (ITS) 2 locus, a region that provides taxonomic discrimination of medically relevant fungi. Each sample yielded an average of 27,553 fungal sequences and *Candida albicans* was the most abundant species identified by sequencing and quantitative PCR (qPCR). Low numbers of *Candida krusei* and *Candida parapsilosis* sequences were observed in several samples, but their presence was detected by species-specific qPCR in only one sample, highlighting a challenge inherent in the study of low-abundance organisms. Overall, the sequencing results revealed that infant fecal samples had fungal diversity comparable to that of bacterial communities in similar-aged infants, which correlated with the relative abundance of *C. albicans*. We conclude that targeted sequencing of fungal ITS2 amplicons in conjunction with qPCR analyses of specific fungi provides an informative picture of fungal community structure in the human intestinal tract. Our data suggests that the infant intestine harbors diverse fungal species and is consistent with prior culture-based analyses showing that the predominant fungus in the infant intestine is *C. albicans*.

## Introduction

Commensal and pathogenic microbes play a fundamental role in human health. Co-development of microbial communities with host metabolism and immunity has significant impact on the risk of disease (e.g. atherosclerosis, obesity, and allergy) throughout a person’s lifespan [1,2,3]. Although the majority of studies to date have focused on associations of bacterial community structure with human diseases, fungi also colonize humans [4], and recent reports highlight the importance of fungal communities in mediating inflammation in the intestine [5,6] and inhibiting disease in the oral cavity of HIV-infected patients [7].

Fungal community profiling by sensitive, specific molecular methods has lagged behind that of bacteria. Shotgun sequencing is hindered by the greater relative abundance of bacterial DNA as compared to that of fungi in most, if not all, human samples. Thus, the majority of investigators studying fungal communities in a high-throughput manner are using DNA-enriching strategies for fungi (reviewed in [8]). These involve using PCR to generate amplicons of regions within the fungal rDNA. Previous studies have almost universally utilized the 454 Life Sciences pyrosequencing platform to study fungal microbiomes; the longer sequences (400–600 bp) generated with this technology are thought to be necessary for accurate identification of fungal taxa. However, the greater sequence read yields of Illumina sequencing platforms (5–10 million reads/flow cell on the MiSeq platform using version 2 chemistry), as compared to 454 pyrosequencing (<1 million reads/run), offer the opportunity to study more samples simultaneously at a lower cost per sample.

Currently, ITS regions of the fungal rDNA are the most utilized targets for generating PCR amplicons for identification of fungal taxa in human samples. Primers targeting ITS1 and ITS2 have been used, with both exhibiting generally comparable accuracies for taxonomic classifications [9], depending on the size of the amplicon and the environmental niche sampled. For example, ITS1 amplicons tend to slightly bias towards identification of Basidiomycetes whereas ITS2 amplicons favor identification of Ascomycetes [10], the latter of which is the taxonomic group containing the majority of human-associated fungi. Recently, the Fungal ITS Workshop Group expressed a preference for using ITS2 over ITS1 [11] because the former region had less variation in read length and more information available regarding secondary structure to inform sequence alignments [12,13]. In addition, when used in pyrosequencing, ITS2 resulted in greater taxonomic diversity and richness than whole ITS [14] and is thought to lead to less PCR bias than ITS1 by virtue of the smaller, more stable size range of ITS2 [10,14]. Further, ITS2 appears to have better sequence representation in databases than ITS1 [15].

In this study, we describe the development and validation of complementary molecular approaches to study fungal community structure in the intestinal tract of infants, a group at particularly high risk for invasive fungal disease and fungal-associated intestinal injury and inflammation [16,17,18]. High-throughput, deep DNA sequencing of amplified ITS2 sequences from the fungal microbiome, using the Illumina MiSeq platform, is used to broadly characterize fungal composition in fecal samples. Additionally, quantitative PCR (qPCR) is employed as a secondary determination of fungal taxa presence in samples and to quantify the abundance of *Candida* species; this yeast is the dominant fungal genus in the human intestine [19,20]. The quantification aspect of our approach has the potential to add important information with respect to *Candida* species colonization because greater intestinal colonization is thought to increase the risk for intestinal injury and invasion by *Candida* species [21,22,23]. In sum, we propose that using a combined amplicon-based sequencing/qPCR approach to fungal community analysis provides new, broader, and corroborative information concerning intestinal fungal microbiomes than analyses using a single methodology.

## Results

### Design and validation of PCR strategy for the identification and quantification of fungi in human fecal samples

**Fig 1:**
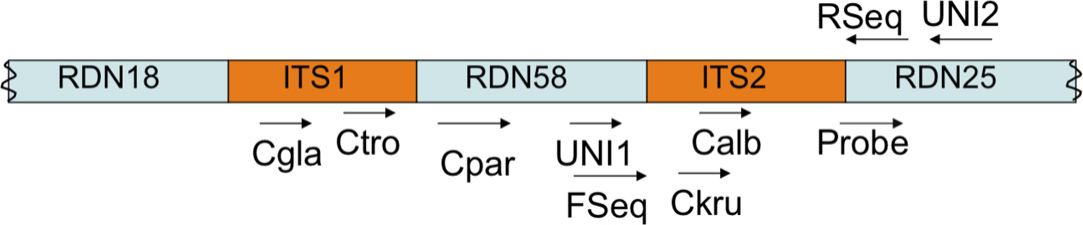
Organization of fungal rDNA locus and regions targeted by oligonucleotide probe and primers. Primers UNI1, UNI2, and Cspecies, and probe are used in the qPCR strategy. Primers Fseq and Rseq are used to generate amplicons for sequencing. Cgla, *C*. *glabrata*; Ctro, *C*. *tropicalis*; Cpar, *C*. *parapsilosis*; Ckru, *C*. *krusei*; Calb, *C*. *albicans*. Locus depiction is not to scale. Primer sequences are given in Table 1.

**Fungal detection.** To screen patient samples for the presence of fungi, we developed a “universal” primer pair (UNI1/UNI2) and oligonucleotide probe that target 18S rDNA loci (Fig. 1 and Table 1) of a broad range of fungi and that do not align to non-fungal sequences, based on *in silico* analysis using NCBI-BLAST (S1–S3 Files). The primer pair and probe selected were able to detect DNA from all nine fungal species available from our laboratory stocks (*Candida albicans*, *Candida glabrata*, *Candida parapsilosis*, *Candida tropicalis*, *Candida krusei*, *Saccharomyces cerevisiae*, *Cryptococcus neoformans*, *Fusarium verticillioides*, and *Neurospora crassa*). Importantly, the primer and probe did not amplify human or bacterial DNA, DNA from sterile water samples, or DNA from a sample from a clean, unused diaper (data not shown) (DNA sources listed in Table 2).

**Table 1:** Primer and probe sequences.

Primer Name	Description	Sequence (5’ to 3’)
UNI1	qPCR Universal Forward	ATGAAGAACGCAGCGAAATGCGATA
UNI2	qPCR Universal Reverse	GTTGGTTTCTTTTCCTCC
Probe	qPCR Universal Probe	/56-FAM/CAATAAGCG/ZEN/GAGGAA/3IABkFQ/
Calb	*C*. *albicans* Forward	CTTTGACAATGGCTTAGGTCTAAC
Cpar	*C*. *parapsilosis* Forward	GTCAACCGATTATTTAATAG [30]
Ckru	*C*. *krusei* Forward	ctggccgagcgaactagact [30]
Ctro	*C*. *tropicalis* Forward	TATTGAACAAATTTCTTTGGTGGC
Cgla	*C*. *glabrata* Forward	TTGTCTGAGCTCGGAGAGAG [30]
JB4728	*C*. *albicans* rDNA Cloning Primer Forward	cgcggatccTCAACGAGGAATTCCTAGTAAG
JB4729	*C*. *albicans* rDNA Cloning Primer Reverse	CCGCTCGAGTTCACTCGCCGCTACTGAGGC
FSeq	Sequencing Forward	ATGCCTGTTTGAGCGTC
RSeq	Sequencing Reverse	CCTACCTGATTTGAGGTC

**Table 2:** Genomic DNA used in this study.

Organism/Cell Type	Reference/Source
*Candida albicans* (SC5314)	[63]
*Candida parapsilosis* (A010)	Clinical Microbiology Laboratories, University of MN
*Candida glabrata* (ATCC 66031)	American Type Culture Collection (Manassas, VA)
*Candida tropicalis* (ATCC 750)	American Type Culture Collection (Manassas, VA)
*Candida krusei* (653)	Gift of P. Magee, University of Minnesota
*Neurospora crassa*	Gift of R. Brambl, University of Minnesota
*Fusarium verticillioides*	Gift of G. May, University of Minnesota
*Cryptococcus neoformans*	Gift of K. Nielsen, University of Minnesota
*Saccharomyces cerevisiae* (AS4)	[64]
*Escherichia coli* (DH5-alpha)	Gift of J. Fuchs, University of Minnesota
Human enterocytes	[65]

Use of a plasmid containing the *C*. *albicans* rDNA target sequence indicated that the UNI1/UNI2 primer pair resulted in a low frequency of false-negative results. When very low quantities (~1 copy) of plasmid were used as template, only 1 of 60 samples failed to produce a PCR product. The remaining 59 samples gave quantification cycle (Cq) values of ~33–35. Thus, we define the limit of detection (LoD) of the primer pair as 1 copy of *C*. *albicans* rDNA. The false-positive rate that was associated with use of UNI1 and UNI2 was also low. For samples containing no fungal DNA (water, human, and bacterial [*E*. *coli*] DNA), ~10% of samples (n > 200) gave positive reactions, similar to what has been reported using an alternative “universal” fungal primer pair [24]. Importantly, the false-positive reactions observed in our study were associated with higher Cq values than those obtained from reactions containing very low quantities (~1 copy of plasmid) of fungal DNA (Cq of 38–40 vs. 33–35). When using the UNI1/UNI2 primer pair in qPCR analyses, we define a Cq ≥ 38 as indicating a sample lacking fungal DNA. The standard curve generated from three separate experiments with this primer pair and dilutions of the *C*. *albicans* rDNA plasmid displayed an excellent amplification profile with an r^2^ > 0.99, efficiency of ~103%, and quantitative dynamic range of 100–1 × 10^9^ rDNA copies per reaction (S1 Fig.). Thus, we have confidence that the UNI1/UNI2 primer pair reliably detects fungi within a given sample and is useful for screening clinical samples for the presence of fungal DNA. Based on these determinations, if a sample yields a Cq signal < 38 in at least 2 of 3 independent replicates, it will be considered to be positive for fungal DNA.

***Candida* species identification and quantification.** *Candida* species are thought to be the major fungal colonizers of the human intestine and cause the majority of fungal disease in immunocompromised patients, especially premature infants [21,22,23]. To distinguish and quantify *Candida* species in fecal samples, we developed a qPCR strategy using species-specific primer pairs targeting 18S rDNA loci of the five most common *Candida* species (*C*. *albicans*, *C*. *parapsilosis*, *C*. *glabrata*, *C*. *tropicalis* and *C*. *krusei*) with respect to human colonization and disease [25] (locations depicted in Fig. 1). Primers specific for *C*. *krusei*, *C*. *parapsilosis*, and *C*. *glabrata* rDNA, designed and published previously for use in conventional PCR ([26], Table 1), were found to accurately identify the appropriate species, and not the other *Candida* species, when used in qPCR experiments. Optimized primers targeting the *C*. *albicans* and *C*. *tropicalis* rDNA loci were newly designed for this study (Table 1) and, likewise, were specific for their corresponding species in qPCR experiments. In addition, no signals were generated with any of the five species-specific primer pairs using non-fungal DNA (*E*. *coli*, human) or samples lacking template DNA (water; material from unused diaper). We also investigated the performance of primer pairs in detecting fungi in simple mock communities. In defined mixes (1:1, 1:10, 10:1) of *C*. *albicans* and *C*. *parapsilosis*, the leading fungal pathogens of infants, both *C*. *albicans*- and *C*. *parapsilosis*-specific primer pairs yielded qPCR results consistent with their respective concentration loads and without evidence of an inhibitory effect from the presence of non-target DNA (S1 Table). In addition, the *C*. *albicans*-specific primer pair readily identified *C*. *albicans* in a mixed culture containing *C*. *albicans*, *N*. *crassa*, *F*. *verticillioides*, and *S*. *cerevisiae*. Further, use of the *Candida* species-specific primers with DNA from fecal samples from infants diagnosed with *C*. *albicans* and *C*. *krusei* pneumonia (tracheal cultures positive) gave qPCR signals for the respective *Candida* species only. This finding provides further evidence of the robustness of our qPCR strategy in clinical samples and is consistent with the idea that disseminated candidiasis results from translocation of prevalent commensal *Candida* species across the intestinal epithelium, as previously proposed by others [21].

**Fig 2:**
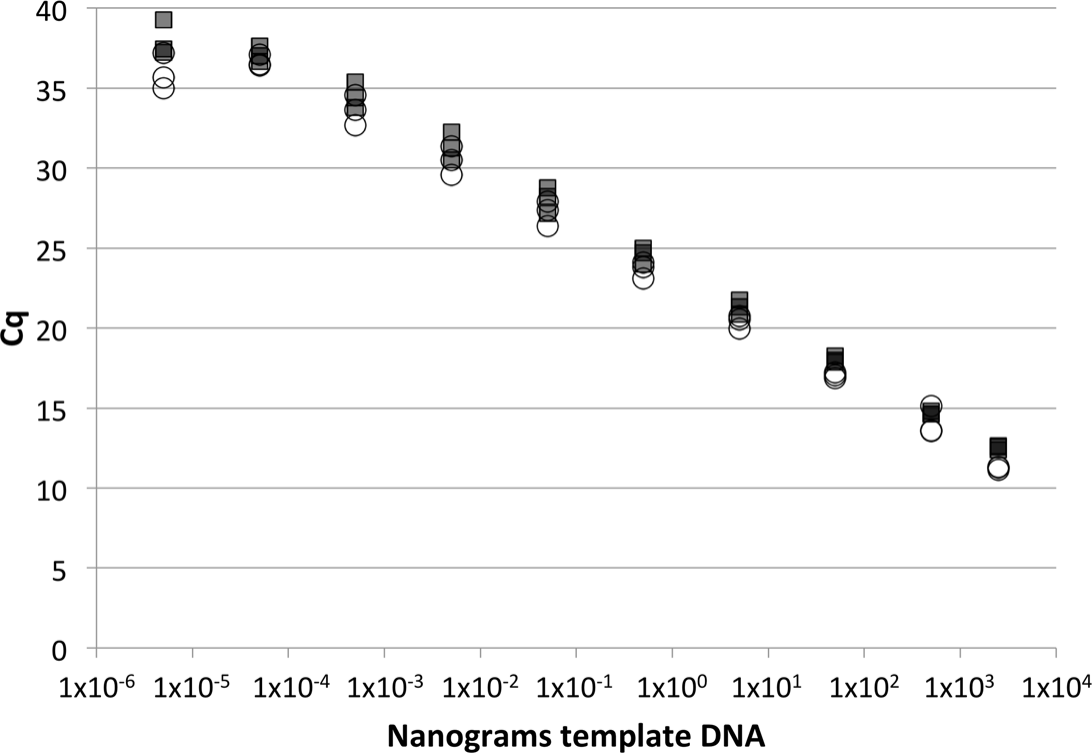
Standard curve amplification plots using *C*. *albicans* genomic DNA as template. Standard curves were generated using 10-fold serial dilutions of DNA with both the universal (UNI1 and UNI2) (circles) and the *C*. *albicans*-specific (squares) primer pairs. n = 3 determinations for each DNA amount and primer tested. r^2^ = 0.999 for the log-linear portion of each curve.

Of all *Candida* species, *C*. *albicans* is the predominant intestinal colonizer and cause of fungal disease in humans [29], thus, knowledge about its colonization characteristics within the larger intestinal fungal community is important to gain. To evaluate the sensitivity and efficiency of qPCR using the *C*. *albicans*-specific primer pair, we generated standard curves using the plasmid containing a single copy of *C*. *albicans* rDNA as the template. The lower limit of quantification (LoQ) using the *C*. *albicans* primer pair, determined visually from the standard curve, was ~10 rDNA copies/reaction (Cq value of 34, S1 Fig.). In contrast, the LoD was ~1 rDNA copy/reaction (S1 Fig.). The false-negative rate was <1% (n = 60) when using very low amounts (~1 copy/reaction) of plasmid. The false-positive rate was higher, with ~10% of non-target samples (i.e. containing no fungal DNA) displaying a qPCR signal (n = 73). Similar to results with the UNI1/UNI2 primer pair, false-positive samples tended to yield higher Cq values (38–40) than samples containing very small amounts (1 copy/reaction) of *C*. *albicans* rDNA (34–36). Thus, for the *C*. *albicans*-specific primer pair, we define a Cq value of <38 as indicating the presence of *C*. *albicans* DNA in the reaction. Further, *C*. *albicans*-specific standard curves displayed a consistent and efficient profile with an r^2^ > 0.99 and an efficiency of ~107%, indicating a high degree of reproducibility and linearity when using different concentrations of plasmid template. Standard curves were also generated that relate qPCR signal to *C*. *albicans* genomic DNA amount (Fig. 2). The quantitative dynamic range, using genomic DNA as template, was estimated to be ~50 fg—2.5 μg of DNA per reaction with an r^2^ value of >0.99 and an efficiency of 103% (Table 3). This equates to a theoretical lower limit of quantification of 3–10 *C*. *albicans* genomes (based on genomic mass of 16.2 fg [27,28]) and a theoretical LoD of one genome. Standard curves generated with cell dilutions rather than purified DNA provided similar cell/genome equivalent values (data not shown). The presence of fecal material resulted in a slight shift of the standard curve to the left (data not shown) indicating that, for feces, DNA extraction techniques may be less efficient and/or that feces may contain PCR inhibitors. Previous studies have estimated the number of rDNA copies in *C*. *albicans* at between 25 and 125 per genome depending upon the strain, with a published average of ~55 copies per genome [29,30]. In comparing the qPCR standard curves generated with cell dilutions to those generated with rDNA plasmid dilutions, we estimated that the average number of rDNA copies per *C*. *albicans* SC5314 cell was 8.3. The observation that our values are lower than the published estimates implies that the laboratory strains used in each study differ with respect to the number of rDNA copies present or, alternatively, that technical variations existed in one or both studies to cause differences in copy number determination.

**Table 3:** qPCR primer sensitivities and reaction efficiencies.

Species DNA	Primer	Efficiency %	r^2^
*C*. *albicans*	Uni1	101.0	0.999
*C*. *albicans*	Calb	103.1	0.999
*C*. *glabrata*	Cgla	112.1	0.998
*C*. *parapsilosis*	Cpar	102.7	0.992
*C*. *tropicalis*	Ctro	111.1	0.994
*C*. *krusei*	Ckru	112.1	0.998

Efficiencies and r^2^ values determined using the log-linear regions of standard curves generated using 10-fold serial dilutions of *Candida* species genomic DNA.

Standard curves generated for *C*. *glabrata*, *C*. *parapsilosis*, *C*. *tropicalis* and *C*. *krusei*, using the appropriate species-specific primer pairs along with dilutions of genomic DNA, gave quantitative dynamic ranges of ~20 fg—1 μg of genomic DNA per reaction, qPCR efficiencies of ~101–112%, and r^2^ values > 0.99 (Table 3). These results indicate that the non-*C*. *albicans* primer pairs are consistent when used with different concentrations of DNA, and that each is able to efficiently generate a PCR product. False-negative rates for each of the non-*albicans* species-specific primers were low (range 0–16%, n ~ 30 for each species) and none of the primers displayed false-positive reactions (0 out of ~30 reactions for each species). The lower LoQ for each primer pair was determined visually from the standard curves and occurred at Cq values of 34 (*C*. *glabrata*), 32 (*C*. *parapsilosis*), 34 (*C*. *tropicalis*), and 33 (*C*. *krusei*), which correlate to ~5–20 fungal cells per reaction, depending on the species examined (S2–S5 Figs.).

### MiSeq fungal sequencing platform development

For sequencing of the fungal microbiome in clinical samples, an oligonucleotide primer pair (FSeq and RSeq) was developed that targeted conserved regions surrounding ITS2 of the fungal rDNA locus (Fig. 1). We estimated, from a recent study [10], that fungal amplicons generated with these primers would generally fit within the size range for 2 × 150 paired-end sequencing by the Illumina MiSeq platform. Forty individual barcodes, optimized to remove bias in Illumina-based library preparations [31], were included in the construction of the forward primer allowing multiplex sequencing of up to forty individual samples per run, with a coverage potential of at least 100,000 sequence reads per sample. This amount of sequence output provided saturable coverage of the fungal population as estimated from previously published studies [32,33] and rarefaction curves generated from the data (S6 Fig.).

Primer sequences were tested *in silico* using NCBI-BLAST against the nr database and both the forward and reverse primers were found to match tens of thousands of unique fungal hits, including medically relevant fungi such as *Candida* species, indicating a wide diversity of putative fungal targets (S4 and S5 Files). The primers were evaluated for their ability to amplify, and be used in sequencing reactions, with DNA isolated from a culture containing only *C*. *albicans*. As expected, 99.97% of the obtained reads were identified as *C*. *albicans*. The next most abundant taxon identified was *C*. *africana*, which has been proposed to be a biovar of *C*. *albicans* (reviewed in [34]).

We hypothesized that early fecal samples passed by infants would be relatively lacking in microbial DNA, as the infant has not had time to be colonized and the intrauterine environment is thought to be sterile. When an early fecal sample in our collection was subjected to sequencing, only 49 reads were obtained, the majority of which were *C*. *albicans*. Consistent with the very low number of sequencing reads, the sample yielded no signals by replicated qPCR when using either the UNI1/UNI2 primer pair or species-specific primer pairs. We used this fecal sample to build a mock fungal community (qPCR-negative fecal sample spiked with moderate amounts of *C*. *albicans*, *S*. *cerevisiae*, *N*. *crassa*, and *F*. *verticillioides*) and analyzed it to evaluate the robustness of the sequencing strategy and bioinformatics pipeline. Thousands of high-quality sequencing reads from each of the species were obtained (data available at the GenBank accession number provided in Methods), consistent with the idea that the primers used to generate sequencing amplicons are not obviously biased toward *C*. *albicans*. As expected, sequences from fungal species not included in the mix were not obtained. Thus, the FSeq and RSeq primers are able to generate specific amplicons from a variety of fungal species and genera that are suitable for analyses by high-throughput sequencing platforms.

### Use of combined qPCR and sequencing strategies to characterize fecal fungal communities

**Fig 3:**
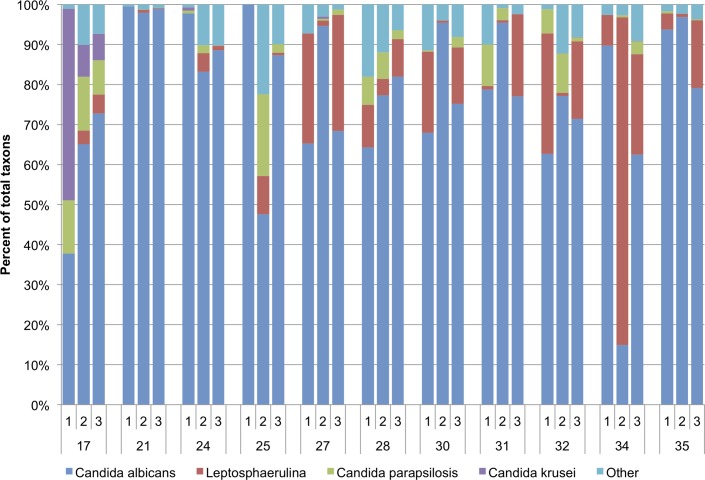
Relative abundances of fungal taxa in infant fecal samples. Sample numbers (each from different infants) are noted on the lower line of the x-axis and the results of triplicate determinations are shown above the sample number for each. The most abundant taxa are indicated on the right-hand side of the graph, with low-abundance taxa (present at < 1.5% mean abundance across all samples) being grouped into the “Other” category. Sequencing reads are displayed as percentages of the total number of reads for each individual sequencing replicate. Full dataset results for all taxa as well as raw percentage values are presented in S2 Table.

**Fig 4:**
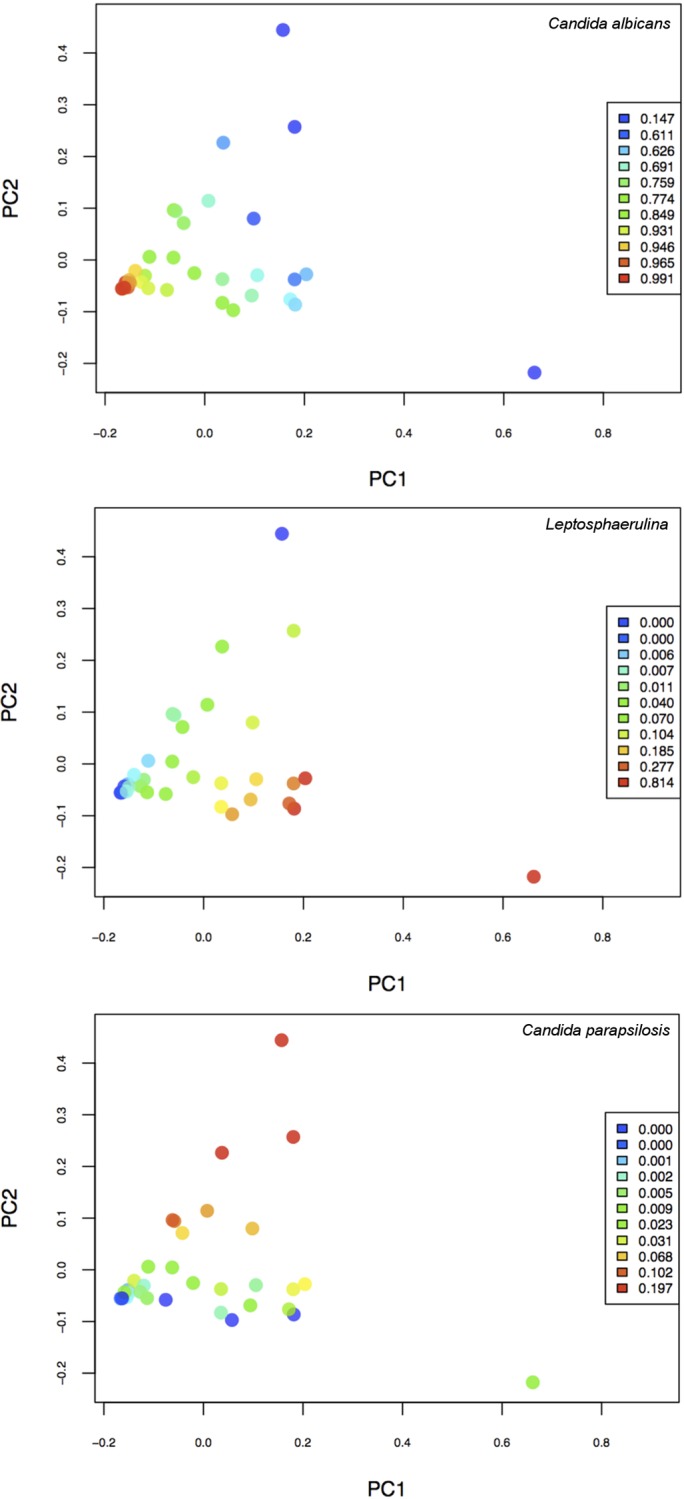
Principal Coordinates analysis of dominant taxa in infant fecal samples. Principal coordinates analysis of Bray-Curtis dissimilarities among the three most abundant taxa (*C*. *albicans*, *Leptosphaerulina*, and *C*. *parapsilosis*) was performed for each sample. Each sample is represented by a dot and is colored based on the abundance (indicated in the legend) of the taxon indicated in the title of the graph. The *C*. *albicans*-*Leptosphaerulina* tradeoff is driving the first (PC1) axis (from bottom to top), and the *C*. *albicans*-*C*. *parapsilosis* tradeoff is driving the second (PC2) axis (from left to right), in all graphs.

The qPCR and sequencing strategies described above were used to evaluate fungal community structure in eleven randomly obtained, anonymous infant fecal samples. All of the samples yielded DNA amplicons using the Fseq/Rseq primer pair. Fungal community structures obtained from analysis of the amplicon sequences are shown in Fig. 3 and S2 Table. The mean Shannon diversity indices of the samples showed a range of values (Table 4). The highest Shannon diversity index was seen in sample 17 (2.25 ± 0.16) and the lowest in sample 21 (0.79 ± 0.22), with standard deviations of all samples ranging from 0.11 to 0.82, indicating variation among replicates. Additionally, we performed principal coordinates analysis using between-sample Bray-Curtis distances of the species-level taxon relative abundances. We identified the three most abundant taxa (*C*. *albicans*, *Leptosphaerulina*, and *C*. *parapsilosis*) across samples, and colored the principal coordinates plot points according to the relative abundances of these taxa in order to visualize covariation between them (Fig. 4). *C*. *albicans*, which is the most abundant, was strongly associated with the first principal axis of variation and thus appeared to drive the variation in the cohort. For example, the least diverse samples tend to be those with the highest *C*. *albicans* abundance (Fig. 4, lower left quadrant of top graph; S7 Fig.).

**Table 4:** Comparison of data from sequencing and qPCR for eleven infant fecal samples.

	Sequencing results*								qPCR results by primer*			
Sample	Mean reads	StDev Reads	Mean Shannon	StDev Shannon	*C*. *albicans*	*C*. *parapsilosis*	*C*. *tropicalis*	*C*. *krusei*	Uni	Calb	Cpar	Ckru
17	2259	2133	2.25	0.16	+++	+++	−−−	+++	+++	+++	+++	+
21	282972	351107	0.79	0.22	+++	+++	++−	+++	+++	+++	+−−	−
24	1764	1917	1.57	0.74	+++	++−	+−−	++−	+++	+−−	−−	−−
25	2087	3443	1.76	0.82	+++	++−	−−−	−−−	+++	++−	−−	−−
27	338	452	1.72	0.20	+++	++−	−−−	+−−	+++	+++	−−	−
28	2248	2509	2.19	0.18	+++	+++	+−−	−−−	+++	+++	−−	−
30	1911	1728	2.03	0.11	+++	+++	++−	−−−	+++	++−	−−	−
31	2406	1616	1.80	0.26	+++	++−	+−−	−−−	+++	+−−	−−	−
32	3016	3651	1.81	0.40	+++	+++	−−−	++−	++−	+++	−−	−
34	3009	2204	1.78	0.38	+++	++−	−−−	+−−	+++	++−	−−	−
35	1078	988	1.55	0.55	+++	++−	−−−	+−−	+++	++−	−−	−

* +, positive qPCR signal (above the LoD) in one replicate sample;-, no qPCR signal (below the LoD). As shown, most determinations were performed in triplicate; some determinations were done in duplicate or singly due to lack of template material.

All samples were positive for fungi (in at least 2 of 3 replicates) by qPCR, using the UNI1/UNI2 primer pair (Table 4). Thus, qPCR and sequencing results generally corroborate each other with respect to fungal presence in the samples. At least 2 of 3 sequencing replicates from all eleven samples showed a majority of sequences identified as *C*. *albicans*. qPCR analyses of the samples using the *C*. *albicans*-specific primer pair corroborated this result, showing the presence of *C*. *albicans* in nine of the samples; the remaining two samples (samples 24 and 31) had signal by qPCR in only 1 of 3 replicates. Only one of the eleven samples (sample 21) yielded a *C*. *albicans* qPCR signal within the range of quantification (1 × 10^6^ cells/g feces); this sample was from the only infant carrying a diagnosis of *Candida* infection (mucocutaneous candidiasis). By comparison, plating sample 21 on fungal growth medium resulted in an estimated cell count of 1 × 10^5^ cells/g feces.

*Candida* species other than *C*. *albicans* were also detected by sequencing, although with less abundance (Fig. 3). *C*. *parapsilosis*, the second most common cause of invasive fungal disease in infants [35], was detected in at least 2 of 3 replicates in all of the samples. However, by qPCR, only one of the eleven samples (sample 17) was positive using the *C*. *parapsilosis* primer pair (Table 4). When the sequencing results were analyzed more closely, the number of *C*. *parapsilosis* sequence reads in the other, qPCR-negative, samples was relatively low (mean of 2.7% of total reads, with a range of 0.8–7.5% within individual samples) as compared to sample 17 (mean of 11.8% of reads). Thus, the lack of complete concordance between qPCR and sequencing results with respect to *C*. *parapsilosis* appears to be associated with its low abundance in the samples. The qPCR result for sample 17 was below the lower LoQ, thus an estimated cell count for *C*. *parapsilosis* could not be determined. This sample was plated on fungal growth medium and grew only *C*. *parapsilosis* (estimated fungal count of 30 cells/g feces), confirmed by qPCR of DNA isolated from colonies using species-specific primers. No other species of fungus grew from sample 17 and no other fecal samples were positive for *C*. *parapsilosis* growth by culturing methods.

*C*. *krusei* was detected by sequencing in four of the eleven samples; only one of these (sample 17) was positive by qPCR (Table 4). The estimated cell count by qPCR was at least 1.6 × 10^3^ cells/g feces; *C*. *krusei* was not isolated by culture-based methods. *C*. *tropicalis* was detected by sequencing in two of the eleven samples; no samples were positive by qPCR (Table 4). *C*. *glabrata* was not detected by either sequencing or qPCR in any of the samples. The other abundant taxon identified by sequencing was *Leptosphaerulina*, which was present in at least 2 of 3 replicates for all eleven samples and ranged between 0.32 and 38.18% of sequence reads (Fig. 3). This taxon has no known medical relevance reported, and members are most commonly identified as plant pathogens [36]. Of note, no fungal species, with the exception of *C*. *albicans* and *C*. *parapsilosis*, grew from any of the fecal samples on the two culture media used, and samples 17 and 21 were the only samples to exhibit fungal growth.

## Discussion

High-throughput amplicon-based genomic profiling is a powerful tool that allows researchers to investigate the role of microbial community characteristics in human health and disease. For fungal genomics, these technologies are not perfect, as evidenced in this study by sequencing and PCR results that are not always concordant with each other. Our data suggest that each genomic analysis platform has advantages and disadvantages and their utility should be decided based on the experimental objectives. The low abundance of fungi in most human samples likely contributes to this technical challenge and, thus, it is important to consider the use of complementary methodologies to corroborate findings, especially when making conclusions about the presence of individual species within a community. For example, by sequencing, all of the samples analyzed in this study harbored low numbers of sequences that mapped to *C*. *parapsilosis*; however, only one of the samples was positive by qPCR using validated, sensitive *C*. *parapsilosis*-specific primers. Thus, no definitive conclusions can be made regarding the presence of *C*. *parapsilosis* in these patient samples without further investigation. Possible reasons for false-negative qPCR results include sequence variation in patient isolates that deviates from the sequences in the database that were used to develop the *C*. *parapsilosis*-specific primers. On the other hand, false-positive sequencing results from high-throughput sequencing methods can occur and have been described in recent literature. Putative mechanisms that have been suggested include low-level contamination, sequence errors introduced during PCR (to generate amplicons) or sequencing, and oxidation of DNA during the library preparation step [37,38,39,40]. The latter two issues have the potential to introduce nucleotide polymorphisms that could affect taxonomic designations. Despite these challenges, we argue that high-throughput amplicon sequencing allows comparisons of fungal diversity and richness that remain meaningful, as the technical issues will likely affect all samples to a similar degree. Overall, when used along with other genomic methods such as qPCR, the benefit of high-throughput sequencing methods is in providing information about the broader intestinal fungal community in a large number of samples simultaneously, at decreased time and monetary costs as compared to traditional cloning coupled with Sanger sequencing methods.

This study also highlights that replication is essential to ensure the robustness of amplicon-based genomic results. We found variation within individual sample determinations for both sequencing and qPCR strategies. Such variation is not unexpected and could represent stochastic sampling effects, as has been described recently for fungal community analyses [8,41]. We suggest that, when possible, investigators consider the inclusion of replicate analyses in their experimental design, especially with regard to low-level colonizers such as fungi. This approach will allow the calculation of means (e.g. for diversity indices) that should provide numbers that are theoretically closer to the “true” values.

All of the patients in this study had evidence of intestinal colonization with fungi, detected by sequencing and confirmed by qPCR using the UNI1/UNI2 primer pair. Fungal communities had a range of diversities (Shannon indices 0.79–2.25) but all included *C*. *albicans* sequences, the majority of which were confirmed by qPCR using *C*. *albicans*-specific primers. This result is in agreement with previous culture and non-culture-based determinations in both adults and children that show *C*. *albicans* as the predominant fungus in the human intestinal tract [37,42,43]. In our small cohort of patients, an infant with mucocutaneous candidiasis (sample 21) exhibited the lowest fungal diversity and the highest amount of *C*. *albicans* colonization. In fact, this was the only sample with an amount of *C*. *albicans* that was within the limits of quantification by qPCR. Further study, with larger numbers of infants, is needed in the future to rigorously test hypotheses regarding associations among factors such as fungal diversity, fungal colonization levels, patient demographic and clinical factors and risks for diseases.

Bacterial diversity of human intestinal samples has been reported to range from 2.54 to 4.63 (Shannon indices) in healthy adults when comparing OTUs clustered at 97% identity [44,45,46] and at less than ~2.5 in healthy infants at 3–4 months of age [47] and in premature infants at ~1 month of age [33]. Thus, the fungal diversity of our infant samples appears to be similar to that reported for bacteria in infant samples. In the latter study of premature infants, eukaryotic communities were also analyzed. Although diversity indices were not reported, *Candida* was the predominant fungal genus in the majority of samples. In contrast to our study, *C*. *quercitrusa* was the most abundant *Candida* species identified by sequencing, although experimental replication was not included and no independent confirmation of the presence of this fungal species was performed. *C*. *quercitrusa* has been most commonly described in association with fruit crops [48] but was recently reported as a cause of three clustered cases of candidemia in China [49].

A greater understanding of intestinal fungal colonization during infancy is important to gain because, not only do fungi represent important opportunistic pathogens, fungi as “benign” commensals likely have a role in training host immune and metabolic programs [50,51]. In addition, the injured or immature intestinal tract is the initial site of entry for many neonatal pathogens [52,53,54], including *C*. *albicans* [55]. Furthermore, life-threatening intestinal diseases such as necrotizing enterocolitis occur exclusively in the infant population and are thought to be associated with intestinal microbes [56,57]. *Candida* infections, in particular, are highly associated with necrotizing enterocolitis and focal intestinal perforations [17,58], although a causal link between intestinal *Candida* and these diseases has not been explored. In adults, intestinal fungi have been implicated as causative agents in inflammatory bowel disease and “leaky gut” [59,60]. Thus, intestinal fungal communities and their interactions with other intestinal microbes and with the host are an important focus for future investigation. Such studies will complement the growing knowledge base about relationships between bacterial microbiomes and disease being generated by the Human Microbiome Project (http://commonfund.nih.gov/hmp/index). Sensitive and specific genomic tools that characterize human fungal communities are essential to initiate these studies. Ultimately, an understanding of how fungal microbiome features are associated with disease will pave the way for the development of clinical strategies that modulate the microbiota to promote human health.

## Materials and Methods

### Fecal sampling and handling

Feces from anonymous infants, collected from diapers that were being discarded, were obtained from a biorepository at the University of Minnesota—Minneapolis. The only clinical information that was collected regarding the samples was the presence or absence of a diagnosis of fungal infection. As no identifying patient data was associated with the samples, this study did not meet the regulatory definition of human subjects research. Fecal samples were obtained from diapers using a sterile tongue depressor, placed into sterile 50 ml conical tubes (Corning Inc., Corning, NY), and stored at -20°C for 1–4 h until transfer to the laboratory for storage at -80°C. Prior to use in experiments, fecal samples were thawed and resuspended as a 25% (w/w) solution in either sterile water or phosphate buffered saline (PBS), pH 7.0. Fecal samples were chosen for analysis only if it was estimated that they contained a sufficient amount for all experiments, including replicate analyses.

### Microbiological techniques

For DNA extraction and standard curve development, *Candida* strains were recovered from -80°C freezer stocks by incubation on Yeast Peptone Dextrose agar [61] at 30°C. An aliquot of yeast obtained with a sterile toothpick was diluted in PCR-grade water and serial dilutions were made for use in generating standard curves for qPCR determinations. To compare results from genomic strategies with those of traditional culture-based techniques, yeast strains and fecal samples were incubated on Sabouraud’s dextrose agar with 34 μg/ml chloramphenicol [62] at 30°C for 72 hrs. In addition, in an attempt to identify and distinguish *Candida* species visually, fecal samples were also plated on ChromAgar Candida (BD, Franklin Lakes, New Jersey) and incubated at 37°C for 48 hrs, per the manufacturer’s instructions.

### DNA sources and isolation

The microbial and human cells used as controls and/or for standard curve development (qPCR) are described in Table 2. *C*. *albicans* and *S*. *cerevisiae* strains, and human cells from which DNA was isolated for this study have been described previously [63,64,65]. For DNA isolation from cell cultures, the MasterPure Yeast DNA Purification Kit (Epicentre, Madison, WI) was used according to the manufacturer’s instructions, except DNA was suspended in 35 μl of PCR-grade water rather than TE. DNA was stored at 4°C until use. For DNA isolation from fecal samples, the UltraClean Fecal DNA Isolation Kit (MO BIO Laboratories, Inc., Carlsbad, CA) was used with 250 μL of thawed, resuspended feces, according to the manufacturer’s instructions except that DNA was eluted into 50 μL of PCR-grade water rather than the elution buffer provided by the manufacturer. DNA was stored at 4°C until use.

### qPCR

The oligonucleotide primers and probe used in the qPCR experiments (Table 1) were synthesized by Integrated DNA Technologies (Coralville, IA) and purified by standard desalting techniques by the manufacturer. qPCR assays were carried out in a white 96-well plate (Roche, Indianapolis, IN). Each reaction consisted of 5 μl of DNA template and 15 μl of master mix solution (3 μl PCR-grade water, 1 μl forward primer from 5 μM stock, 1 μl reverse primer from 5 μM stock, 0.3 μl probe from 3 μM stock, and 10 μl LightCycler 480 Probes Master, 2× concentration (Roche, includes Taq polymerase), which yields a total volume of 15.3 μl, only 15 μl of which is used for the PCR) for a final reaction volume of 20 μl. Plates were sealed with LightCycler 480 Sealing Foil (Roche). Reactions were performed using a Roche LightCycler 480 instrument with LightCycler 480 software (release 1.5.0 SP3) using the following program: Step 1: (pre-incubation) 95°C for 10 minutes; Step 2: (amplification) 95°C for 20 sec., 52°C for 20 sec., signal acquisition using a FAM (465–510 nm) filter, 72°C for 30 sec, entire step 2 repeated for 45 total cycles; Step 3: 40°C for 30 sec. Analysis was performed using the same Roche LightCycler software package as used to perform the reaction. The Minimum Information for the Publication of Quantitative Real-Time PCR Experiments (MIQE) Guidelines [66] were followed to validate the primers we designed for use in qPCR.

To evaluate the sensitivities and efficiencies of the universal and *C*. *albicans*-specific primers, a plasmid containing a copy of the *C*. *albicans* rDNA locus was constructed as follows. pBluescript II SK+ (Invitrogen, Carlsbad, CA) was digested using *Bam*H1 and *Xho*1. The rDNA locus of *C*. *albicans* strain SC5314 was amplified from genomic DNA using primers JB4728 and JB4729, designed to include *Bam*H1 and *Xho*I sites, respectively. The product was digested with *Bam*H1 and *Xho*1 and ligated to the digested pBluescript II SK+ to generate p2330.

### Standard curve generation and quantitative analyses

For generation of standard curves that relate fungal DNA amount to PCR cycle time (Cq), fungal DNA was serially and decimally diluted. Analysis of standard curves was performed by first constructing regression models for the log-linear ranges of data for each curve. The regression models yielded values for validation of each curve/primer pair being tested, including the r^2^ value, the slope, and the y-intercept of the line of best fit, thus allowing for the derivation of equations to quantify fungal/DNA amounts for a given Cq value. Additionally, the slopes from the linear regressions were used to calculate qPCR efficiencies for each standard curve generated using efficiency = 10^(-1/slope) − 1^. To evaluate the efficiency of qPCR when fecal samples, rather than isolated fungal DNA, is used, standard curves were also generated using dilutions (10-fold) of fungal cells (quantified microscopically by counting on a hemocytometer) spiked into a fecal samples negative for fungus, as determined by PCR. DNA was isolated using the UltraClean Fecal DNA Isolation Kit (MO BIO Laboratories, Inc.) according to the manufacturers instructions.

### High-throughput sequencing

DNA from fecal samples was amplified by PCR using primers that target conserved regions surrounding the ITS2 region of the fungal rDNA locus (Table 1). Each forward primer was barcoded with one of forty unique 6-mer oligonucleotide sequences in order to distinguish individual samples from each other during the analysis. PCR solutions consisted of 97.5 μl H_2_O, 21 μl 25 mM MgCl_2_, 15 μl 10× PCR buffer, 6 μl 10 mM dNTPs, 3 μl 100 mM forward primer, 3 μl 100 mM reverse primer, 3 μl of sample DNA, and 1.5 μl of Taq DNA polymerase (New England Biolabs, Ipswich, MA). The 150 μl final volume was then split into three 50 μl aliquots, loaded into the thermocycler (Applied Biosystems, Foster City, CA) and subjected to the following thermocycler program: Initial denaturation stage of 94°C for 5 min; a 35-cycle amplification stage consisting of a denaturation step at 94°C for 30 sec, an annealing step at 55°C for 30 seconds, and an extension step of 72°C for 15 sec; and a final extension stage of 72°C for 10 min. Resulting products from the same starting sample were pooled and 10 μl were run on an agarose gel to evaluate for the presence of amplicons. The remaining 140 μl of product were cleaned using the QIAQuick PCR purification kit (QIAGEN, Germantown, MD), following the manufacturer’s instructions, except that DNA was eluted into 30 μl of PCR-grade water instead of the buffer supplied by the manufacturer. Cleaned amplicons were then quantitated with a Qubit 2.0 fluorometer (Invitrogen, Eugene, OR) using the Qubit dsDNA HS Assay Kit (Invitrogen). Equal concentrations of amplicons from each individual fecal sample were pooled into a final solution and submitted for Illumina library construction using the TruSeq Nano kit (Illumina, San Diego, CA) and sequencing on an Illumina MiSeq high-throughput sequencing platform using the 2 × 150 bp paired-end version 2 MiSeq Reagent Kit (Illumina) by the University of Minnesota Genomics Center. Sequencing data has been deposited into GenBank at the NCBI under accession number SRP045827.

### Development of fungal-specific ITS2 sequence databases

Because of computational limits inherent with analyzing large amounts of sequencing data, customized fungal-specific ITS2 taxonomy and alignment databases needed to be constructed, similar to databases already publically available for bacterial taxons (e.g., Greengenes [67]). As a starting point, the fungal database maintained by Henrik Nilsson (http://www.emerencia.org/fungalitspipeline.html, September 28, 2012 update) was obtained. The resulting file was processed to construct a consensus fungal database using the UCLUST command in the USEARCH software suite (usearch_i86linux32 v6.0.307, [68]) with an identity threshold of 0.9. A fungal ITS2 alignment database was then generated using the MUSCLE program (muscle3.8.31_i86linux32, [69]). Template and taxonomy database files for use with the Mothur software suite were generated by modifying the hash.txt and fungalITSdatabaseID.fasta files from the Nilsson database using a custom-written AppleScript (S6 File). Species-level information was manually added into the database for the *Candida* and *Cryptococcus* genera using sequence data from GenBank [70].

### Sequence analysis using Mothur

Data files resulting from the sequencing of fecal samples were obtained from the University of Minnesota Genomics Center. Sequences were trimmed to 120 bp in order to improve 3’ sequence quality. Paired ends were then joined using the fastq-join program, which is part of the ea-utils software package (v. 1.1.2–484, (http://code.google.com/p/ea-utils/) [71]). Subsequent processing and analysis steps were done using the Mothur software suite [72]. Sequences were screened based on quality scores, with window sizes of 50 requiring average scores of 30 or greater to remain; sequences failing quality scores in any window were removed entirely from the dataset. Sequences were also screened for the presence of one of the defined barcodes and sorted into groups as indicated. Alignment was then performed using the previously described fungal ITS2 alignment database as a reference. Sequences were then screened for those with start points, end points, and minimum lengths that matched 90% of the sequences in the population. For clustering and operational taxonomic unit (OTU) analyses, sequence alignments were filtered and screened for unique sequences to reduce computational complexity. Sequences were then pre-clustered with a maximum difference of one mismatch to help reduce sequencing errors [73]. Classification was performed using the custom-generated fungal ITS2 taxonomy and template databases referenced above. Distance calculations were performed as a precursor to OTU clustering using a cutoff of 0.15, and then sequences were clustered into OTUs with a cutoff of 0.03 using the furthest neighbor method. Shannon diversity scores were calculated using Mothur following all processing steps. OTUs containing fewer than ten total reads across all samples were eliminated. Principal components analysis of the Bray-Curtis distances for the three dominant taxa was performed using the *plot_gradients*.*r* command in the MWAS package (https://github.com/danknights/mwas).

## Supporting Information

S1 Fig *C*. *albicans* rDNA standard curves. Ten-fold serial dilutions of a plasmid containing a single copy of the *C*. *albicans* rDNA region were made and subjected to qPCR using the universal (circles) and Calb (squares) primers to determine the limits of detection, log-linear region of amplification, and limits of quantification (see Results). n = 3 for each DNA amount and for each primer pair. For the log-linear region of each curve, the r^2^ values were calculated to be 0.998 (Uni) and 0.997 (Calb).
(TIF)

S2 Fig *C*. *glabrata* DNA standard curves. Ten-fold serial dilutions of *C*. *glabrata* genomic DNA were made and subjected to qPCR using the Cgla primers to determine the limit of detection, log-linear region of amplification, and limits of quantification (see Results). n = 3 for each DNA amount. For the log-linear region of the curve, r^2^ = 0.998.
(TIF)

S3 Fig *C*. *parapsilosis* DNA standard curves. 10-fold serial dilutions of *C*. *parapsilosis* genomic DNA were made and subjected to qPCR using the Cpar primers to determine the limit of detection, log-linear region of amplification, and limits of quantification (see Results). n = 3 for each DNA amount. For the log-linear region of the curve, r^2^ = 0.992.
(TIF)

S4 Fig *C*. *tropicalis* DNA standard curves. Ten-fold serial dilutions of *C*. *tropicalis* genomic DNA were made and subjected to qPCR using the Ctro primers to determine the limit of detection, log-linear region of amplification, and limits of quantification (see Results). n = 3 for each DNA amount. For the log-linear region of the curve, r^2^ = 0.994.
(TIF)

S5 Fig *C*. *krusei* DNA standard curves. Ten-fold serial dilutions of *C*. *krusei* genomic DNA were made and subjected to qPCR using the Ckru primers to determine the limit of detection, log-linear region of amplification, and limits of quantification (see Results). n = 3 for each DNA amount. For the log-linear region of the curve, r^2^ = 0.998.
(TIF)

S6 Fig Rarefaction curves for fungal ITS2 sequences. Curves are identified by infant (first number) and replicates are colored the same.
(TIF)

S7 Fig Correlation analysis of *C*. *albicans* abundance and fungal diversity of fecal samples. The mean values of replicates for each variable were used to generate the plot for each sample.
(TIF)

S1 File Forward Primer UNI1 BLAST results. (TXT)

S2 File qPCR probe BLAST results. (TXT)

S3 File Reverse Primer UNI2 BLAST results. (TXT)

S4 File Forward primer FSeq BLAST results. (TXT)

S5 File Reverse primer RSeq BLAST results. (TXT)

S6 File AppleScript to create mothur-compatible hash.txt and fungalITSdatabaseID.fasta files. (CPT)

S1 Table Species-specific qPCR primers accurately differentiate species when two *Candida* species (*C*. *albicans*, Ca; *C*. *parapsilosis*, Cp) are present in mixed cultures. (DOCX)

S2 Table Complete taxonomic compositions (given as percentages of sequence reads) from sequencing. (PDF)
