# Supplementary figures and images for: Complementary Amplicon-Based Genomic Approaches for the Study of Fungal Communities in Humans
Source: PLoS One. 2015 Feb 23;10(2):e0116705. doi: 10.1371/journal.pone.0116705 (PMC4338280; doi:10.1371/journal.pone.0116705)

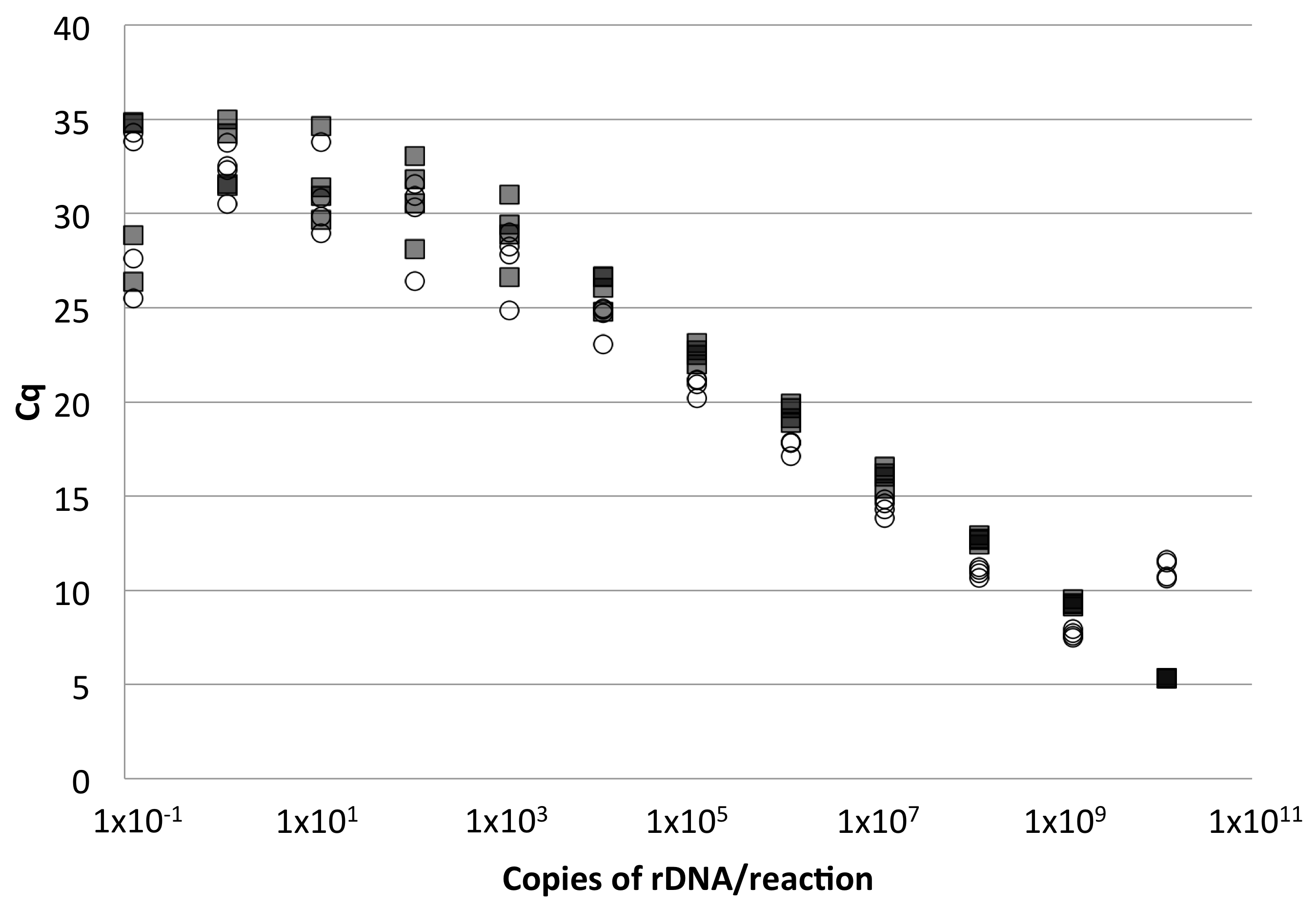

Supplement: S1 Fig — Ten-fold serial dilutions of a plasmid containing a single copy of the C. albicans rDNA region were made and subjected to qPCR using the universal (circles) and Calb (squares) primers to determine the limits of detection, log-linear region of amplification, and limits of quantification (see Results). n = 3 for each DNA amount and for each primer pair. For the log-linear region of each curve, the r2 values were calculated to be 0.998 (Uni) and 0.997 (Calb). (TIF) [file pone.0116705.s001.tif]

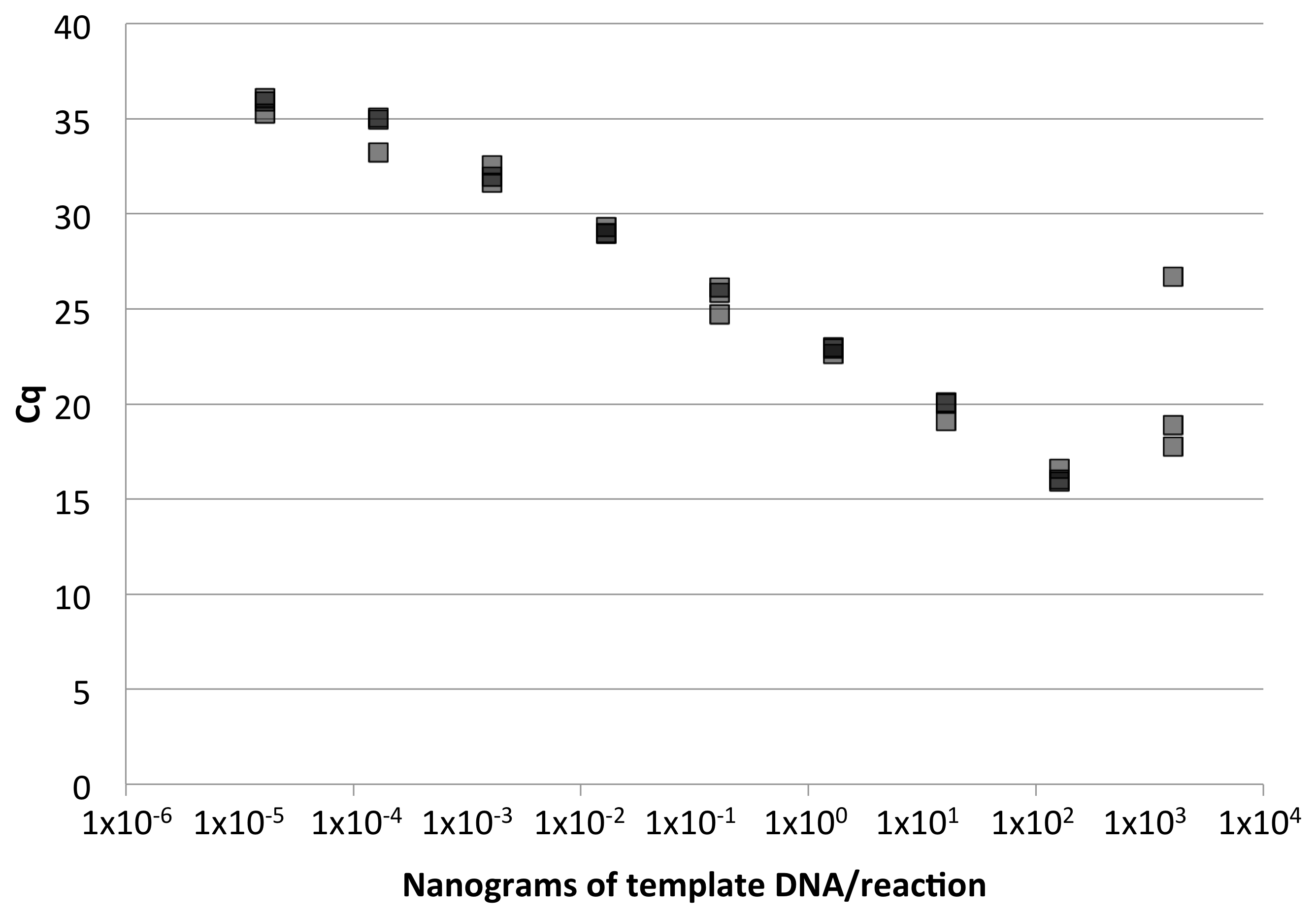

Supplement: S2 Fig — Ten-fold serial dilutions of C. glabrata genomic DNA were made and subjected to qPCR using the Cgla primers to determine the limit of detection, log-linear region of amplification, and limits of quantification (see Results). n = 3 for each DNA amount. For the log-linear region of the curve, r2 = 0.998. (TIF) [file pone.0116705.s002.tif]

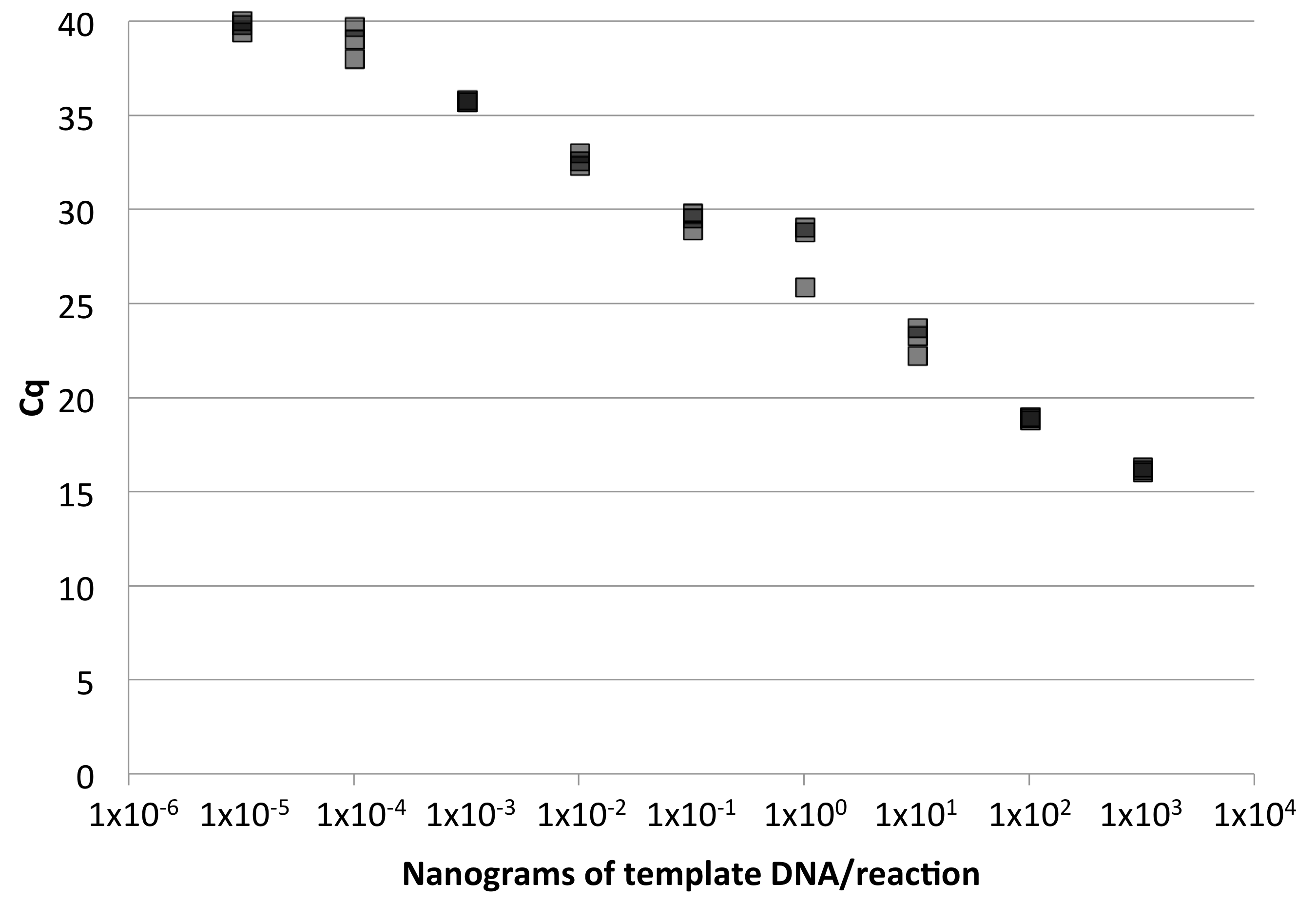

Supplement: S3 Fig — 10-fold serial dilutions of C. parapsilosis genomic DNA were made and subjected to qPCR using the Cpar primers to determine the limit of detection, log-linear region of amplification, and limits of quantification (see Results). n = 3 for each DNA amount. For the log-linear region of the curve, r2 = 0.992. (TIF) [file pone.0116705.s003.tif]

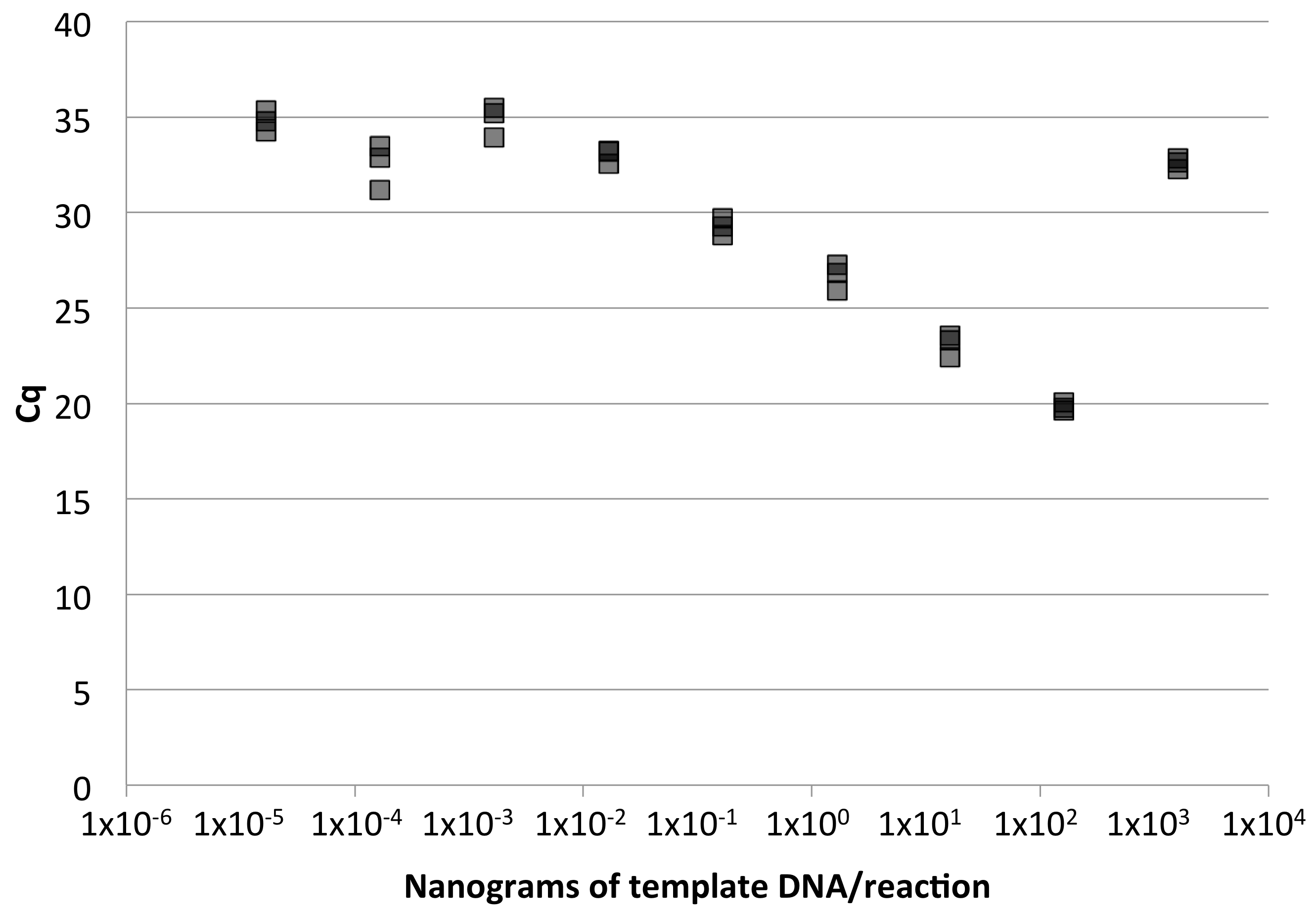

Supplement: S4 Fig — Ten-fold serial dilutions of C. tropicalis genomic DNA were made and subjected to qPCR using the Ctro primers to determine the limit of detection, log-linear region of amplification, and limits of quantification (see Results). n = 3 for each DNA amount. For the log-linear region of the curve, r2 = 0.994. (TIF) [file pone.0116705.s004.tif]

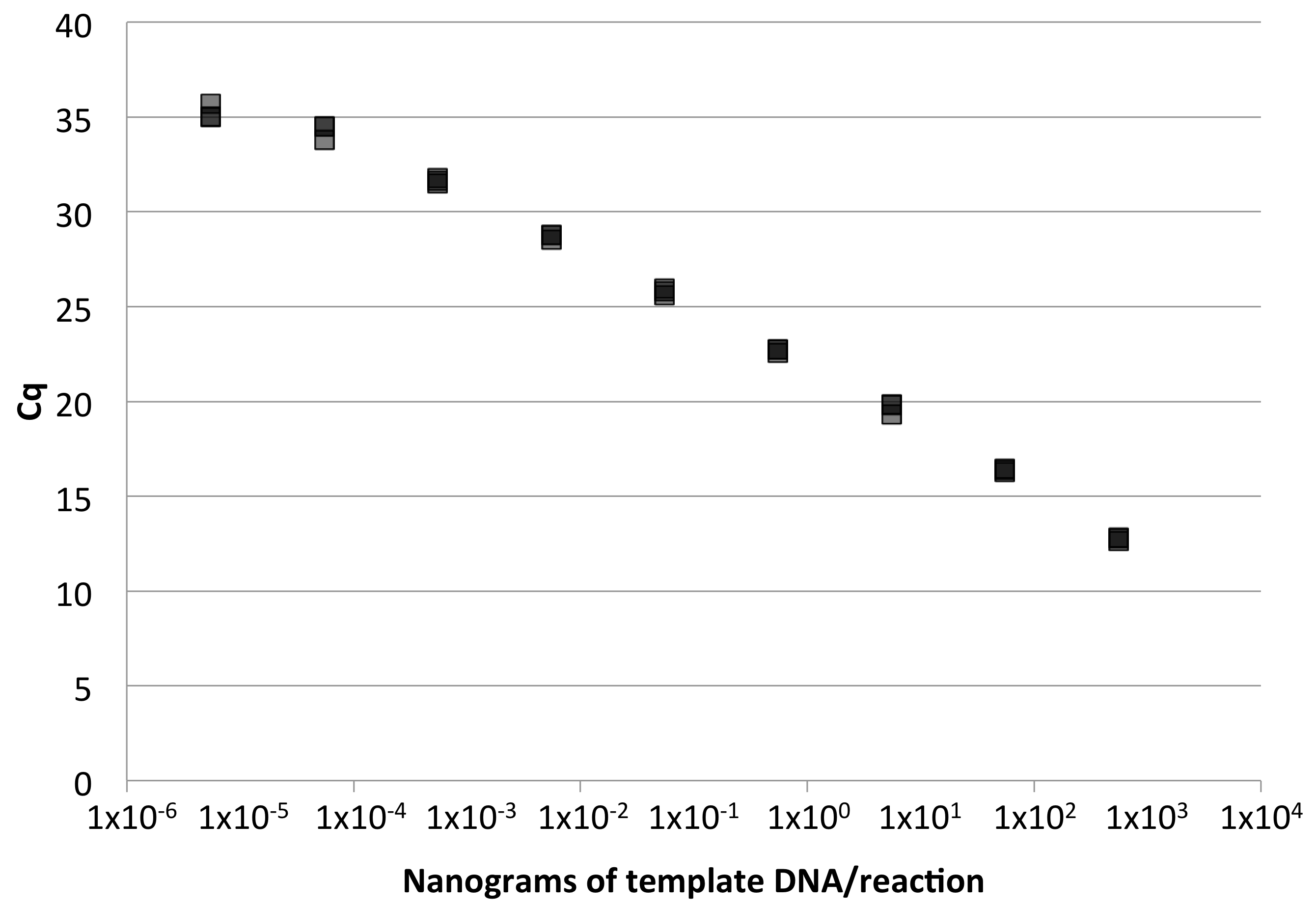

Supplement: S5 Fig — Ten-fold serial dilutions of C. krusei genomic DNA were made and subjected to qPCR using the Ckru primers to determine the limit of detection, log-linear region of amplification, and limits of quantification (see Results). n = 3 for each DNA amount. For the log-linear region of the curve, r2 = 0.998. (TIF) [file pone.0116705.s005.tif]

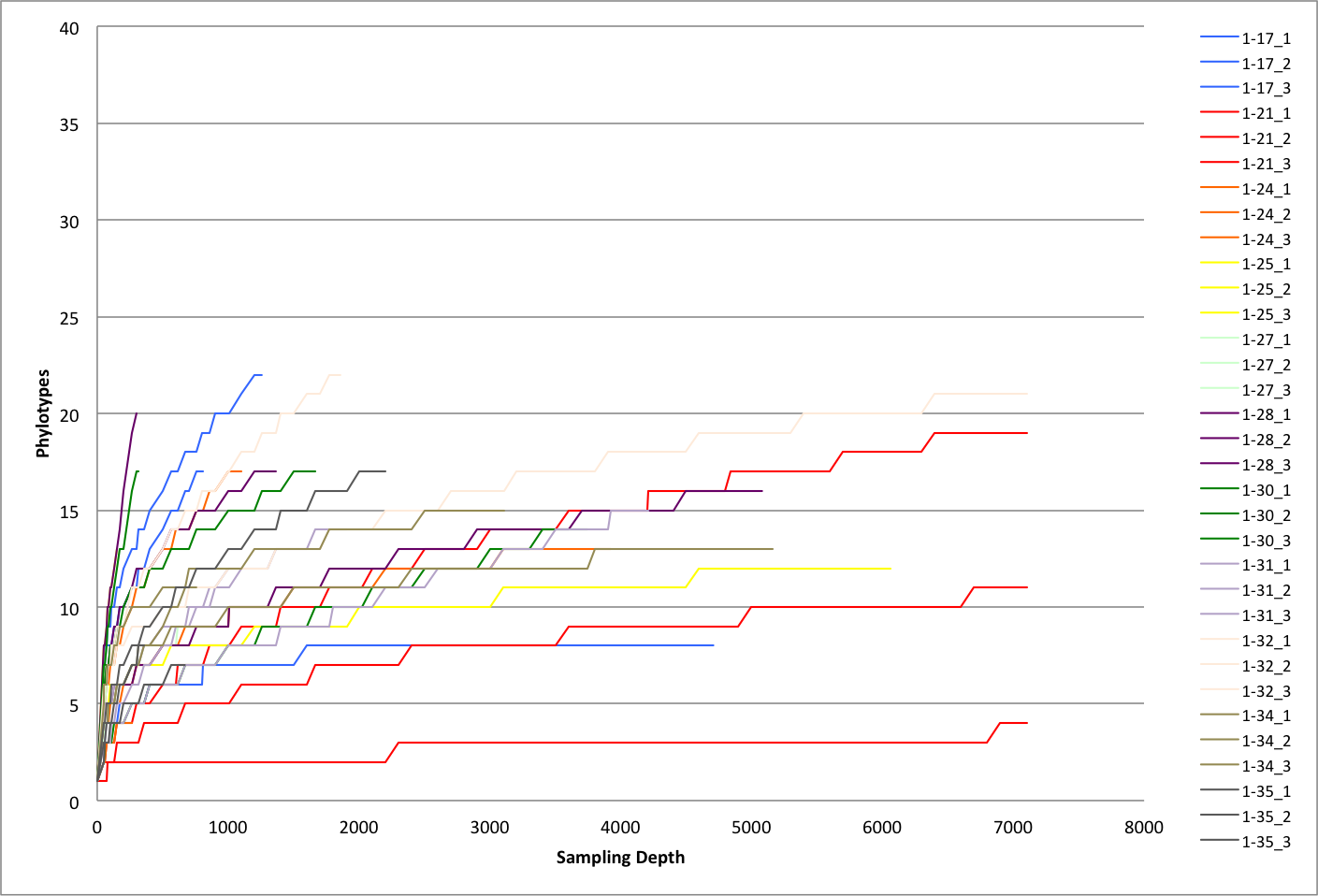

Supplement: S6 Fig — Curves are identified by infant (first number) and replicates are colored the same. (TIF) [file pone.0116705.s006.tif]

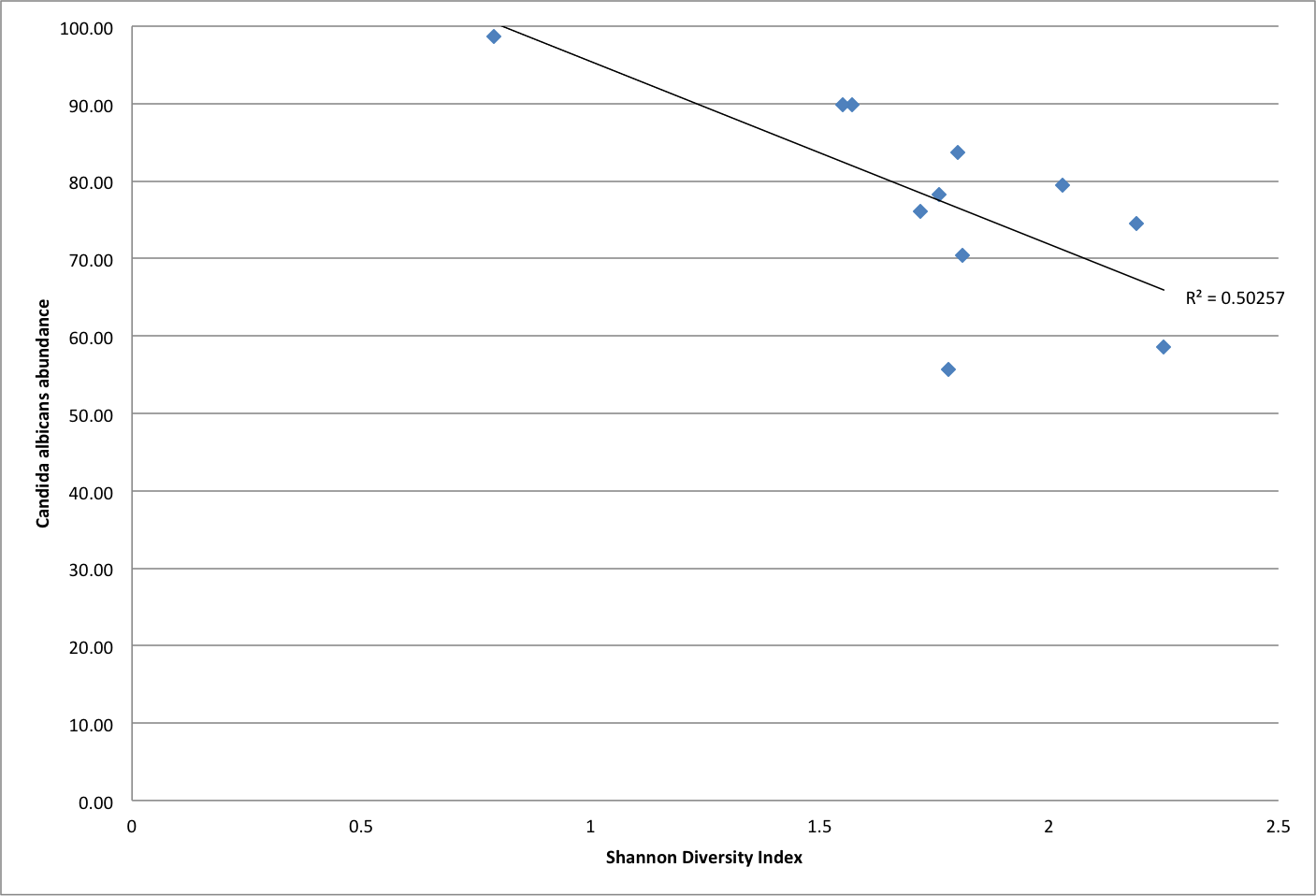

Supplement: S7 Fig — The mean values of replicates for each variable were used to generate the plot for each sample. (TIF) [file pone.0116705.s007.tif]
