# Supplementary material for: Complementary Amplicon-Based Genomic Approaches for the Study of Fungal Communities in Humans
Source: PLoS One. 2015 Feb 23;10(2):e0116705. doi: 10.1371/journal.pone.0116705 (PMC4338280; doi:10.1371/journal.pone.0116705)
Supplement: S1 Table — (DOCX) [file pone.0116705.s014.docx]

**Table S1.** Species-specific qPCR primers accurately differentiate species when two *Candida* species (*C. albicans*, Ca; *C. parapsilosis*, Cp) are present in mixed cultures.

| **Actual Ca cells** | **Actual Cp cells** | **Mean Cq Calb** | **Mean Cq Cpar** | **Calculated Ca cells** | **Calculated Cpar cells** |
| --- | --- | --- | --- | --- | --- |
| 1.00 × 10^6^ | 0 | 22.29 | No signal | 3.91 × x10^4^ | 0 |
| 0 | 1.00 × x10^6^ | No signal | 20.475 | 0 | 1.50 × x10^4^ |
| 1.00 × x10^6^ | 1.00 × x10^6^ | 22.08 | 20.385 | 4.51 × x10^4^ | 1.59 × x10^4^ |
| 1.00 × x10^6^ | 1.00 × x10^5^ | 22.16 | 24.09 | 4.30 × x10^4^ | 1.54 × x10^3^ |
| 1.00 × x10^5^ | 1.00 × x10^6^ | 26.01 | 20.7 | 2.97 × x10^3^ | 1.30 × x10^4^ |
