# Supplementary material for: Complementary Amplicon-Based Genomic Approaches for the Study of Fungal Communities in Humans
Source: PLoS One. 2015 Feb 23;10(2):e0116705. doi: 10.1371/journal.pone.0116705 (PMC4338280; doi:10.1371/journal.pone.0116705)
Supplement: S2 Table — (PDF) [file pone.0116705.s015.pdf]

Table S2. Complete taxonomic compositions (given as percentages of sequence reads) from sequencing.

Sample	17			21			24			25			27			28			30			31			32			34			35		
Replicate	1	2	3	1	2	3	1	2	3	1	2	3	1	2	3	1	2	3	1	2	3	1	2	3	1	2	3	1	2	3	1	2	3
Candida albicans	37.78	65.11	72.78	99.41	97.90	98.87	97.70	83.18	88.55	100.00	47.62	87.27	65.22	94.63	68.42	64.31	77.29	81.97	67.97	95.43	75.14	78.75	95.39	77.00	62.65	77.12	71.41	89.75	14.95	62.46	93.73	96.87	79.08
Leptosphaerulina	0.00	3.37	4.69	0.00	0.74	0.21	0.00	4.70	1.05	0.00	9.52	0.66	27.54	1.31	28.95	10.60	4.09	9.32	20.26	0.52	14.14	0.87	0.70	20.55	30.12	0.81	19.37	7.65	81.80	25.08	3.99	0.78	16.99
Candida parapsilosis	13.30	13.49	8.54	0.01	0.12	0.11	0.77	1.88	0.16	0.00	20.41	2.09	0.00	0.48	1.32	7.07	6.64	2.27	0.33	0.03	2.60	10.38	2.99	0.00	6.02	9.85	0.95	0.00	0.58	3.27	0.57	0.00	0.23
Candida krusei	47.84	7.91	6.62	0.01	0.01	0.00	0.77	0.00	0.05	0.00	0.00	0.00	0.00	0.48	0.00	0.00	0.00	0.00	0.00	0.00	0.00	0.00	0.00	0.00	0.00	0.01	0.11	0.00	0.00	0.03	0.00	0.00	0.05
Cladosporium	0.86	0.00	2.01	0.00	0.10	0.04	0.00	0.56	6.09	0.00	0.00	2.16	1.45	1.07	1.32	13.43	0.10	0.98	0.65	0.03	0.06	3.24	0.02	0.57	1.20	1.62	4.24	0.14	1.32	5.62	0.28	0.00	1.50
Alternaria	0.00	4.54	0.17	0.00	0.03	0.01	0.00	8.27	3.61	0.00	1.36	0.02	1.45	1.91	0.00	0.71	0.02	0.38	0.33	3.27	0.25	6.44	0.70	0.09	0.00	0.01	0.45	1.64	0.02	1.32	0.28	0.00	0.05
Coniothyrium	0.00	3.24	0.00	0.00	0.01	0.01	0.00	0.19	0.00	0.00	14.97	2.97	1.45	0.00	0.00	0.00	2.57	0.76	0.00	0.00	0.19	0.00	0.02	1.13	0.00	0.04	0.28	0.68	0.40	0.10	0.00	0.00	0.09
Chalastospora	0.00	0.00	0.75	0.00	0.01	0.08	0.00	0.19	0.00	0.00	0.68	0.02	0.00	0.12	0.00	0.71	9.23	1.29	5.56	0.00	0.06	0.11	0.00	0.00	0.00	0.01	0.06	0.00	0.00	0.79	0.00	0.00	0.09
Phaeosphaeria	0.00	0.13	0.00	0.00	0.02	0.01	0.00	0.00	0.00	0.00	0.00	4.60	2.90	0.00	0.00	0.35	0.00	1.21	0.00	0.03	1.18	0.00	0.00	0.09	0.00	5.96	0.17	0.00	0.02	0.00	0.00	0.00	1.40
Candida africana	0.22	0.13	0.17	0.56	0.83	0.61	0.77	0.28	0.21	0.00	0.00	0.20	0.00	0.00	0.00	0.71	0.06	0.38	0.98	0.41	0.37	0.05	0.12	0.28	0.00	1.23	0.39	0.00	0.06	0.50	0.85	0.47	0.37
Peyronellaea	0.00	0.00	0.17	0.00	0.09	0.00	0.00	0.00	0.03	0.00	0.00	0.00	0.00	0.00	0.00	0.00	0.00	1.06	0.65	0.00	1.43	0.11	0.02	0.19	0.00	2.70	0.06	0.00	0.66	0.83	0.28	0.00	0.00
Phaeococcomyces	0.00	0.13	0.00	0.00	0.00	0.00	0.00	0.56	0.00	0.00	0.68	0.00	0.00	0.00	0.00	0.00	0.00	0.00	0.00	0.00	3.78	0.00	0.00	0.00	0.00	0.00	0.00	0.00	0.00	0.00	0.00	0.00	0.00
Ceratocystis	0.00	0.00	0.00	0.00	0.03	0.00	0.00	0.00	0.00	0.00	4.76	0.00	0.00	0.00	0.00	0.00	0.00	0.00	0.00	0.00	0.06	0.00	0.00	0.00	0.00	0.00	0.00	0.00	0.00	0.00	0.00	0.00	0.00
Periconia	0.00	0.13	4.02	0.00	0.00	0.00	0.00	0.00	0.00	0.00	0.00	0.00	0.00	0.00	0.00	0.00	0.00	0.00	0.00	0.00	0.00	0.00	0.00	0.00	0.00	0.00	0.00	0.00	0.00	0.00	0.00	0.00	0.00
Candida tropicalis	0.00	0.00	0.00	0.00	0.08	0.00	0.00	0.00	0.05	0.00	0.00	0.00	0.00	0.00	0.00	0.00	0.00	0.08	3.27	0.03	0.00	0.05	0.00	0.00	0.00	0.00	0.00	0.00	0.00	0.00	0.00	0.00	0.00
Aureobasidium	0.00	1.69	0.00	0.00	0.01	0.00	0.00	0.00	0.00	0.00	0.00	0.00	0.00	0.00	0.00	1.77	0.00	0.00	0.00	0.00	0.00	0.00	0.00	0.00	0.00	0.00	0.00	0.00	0.00	0.00	0.00	0.00	0.00
Cryptococcus luteolus	0.00	0.00	0.08	0.00	0.00	0.00	0.00	0.09	0.00	0.00	0.00	0.00	0.00	0.00	0.00	0.00	0.00	0.00	0.00	0.00	0.00	0.00	0.00	0.00	0.00	0.01	1.95	0.00	0.00	0.00	0.00	0.00	0.00
Dendryphiella	0.00	0.00	0.00	0.00	0.00	0.00	0.00	0.09	0.00	0.00	0.00	0.00	0.00	0.00	0.00	0.00	0.00	0.00	0.00	0.00	0.00	0.00	0.00	0.00	0.00	0.00	0.00	0.00	0.00	0.00	0.00	1.88	0.00
Davidiella	0.00	0.13	0.00	0.00	0.01	0.00	0.00	0.00	0.00	0.00	0.00	0.00	0.00	0.00	0.00	0.00	0.00	0.08	0.00	0.00	0.62	0.00	0.00	0.00	0.00	0.01	0.50	0.14	0.04	0.00	0.00	0.00	0.14
Dothidea	0.00	0.00	0.00	0.00	0.00	0.00	0.00	0.00	0.21	0.00	0.00	0.00	0.00	0.00	0.00	0.00	0.00	0.23	0.00	0.25	0.00	0.00	0.00	0.00	0.00	0.61	0.00	0.00	0.00	0.00	0.00	0.00	0.00
Pichia	0.00	0.00	0.00	0.00	0.00	0.05	0.00	0.00	0.00	0.00	0.00	0.00	0.00	0.00	0.00	0.35	0.00	0.00	0.00	0.03	0.00	0.00	0.00	0.00	0.00	0.00	0.00	0.00	0.00	0.00	0.00	0.00	0.00
Ascomycota	0.00	0.00	0.00	0.00	0.00	0.00	0.00	0.00	0.00	0.00	0.00	0.00	0.00	0.00	0.00	0.00	0.00	0.00	0.00	0.00	0.12	0.00	0.00	0.09	0.00	0.00	0.00	0.00	0.14	0.00	0.00	0.00	0.00
Lachnum	0.00	0.00	0.00	0.00	0.01	0.00	0.00	0.00	0.00	0.00	0.00	0.00	0.00	0.00	0.00	0.00	0.00	0.00	0.00	0.00	0.00	0.00	0.02	0.00	0.00	0.00	0.06	0.00	0.00	0.00	0.00	0.00	0.00
Cryptococcus victoriae	0.00	0.00	0.00	0.00	0.01	0.00	0.00	0.00	0.00	0.00	0.00	0.00	0.00	0.00	0.00	0.00	0.00	0.00	0.00	0.00	0.00	0.00	0.00	0.00	0.00	0.00	0.00	0.00	0.00	0.00	0.00	0.00	0.00
Trimmatostroma	0.00	0.00	0.00	0.00	0.00	0.00	0.00	0.00	0.00	0.00	0.00	0.00	0.00	0.00	0.00	0.00	0.00	0.00	0.00	0.00	0.00	0.00	0.00	0.00	0.00	0.00	0.00	0.00	0.00	0.00	0.00	0.00	0.00
Thelebolus	0.00	0.00	0.00	0.00	0.00	0.00	0.00	0.00	0.00	0.00	0.00	0.00	0.00	0.00	0.00	0.00	0.00	0.00	0.00	0.00	0.00	0.00	0.00	0.00	0.00	0.00	0.00	0.00	0.00	0.00	0.00	0.00	0.00
Total	100	100	100	100	100	100	100	100	100	100	100	100	100	100	100	100	100	100	100	100	100	100	100	100	100	100	100	100	100	100	100	100	100
